# Wooden Penetrating Pelvic Trauma Passing the Foramen Ischiadicum

**DOI:** 10.1155/2019/5834129

**Published:** 2019-12-26

**Authors:** Benedikt Ruben Abel, Heinz-Lothar Meyer, Roman Müller, Katharina Henze, Marcel Dudda, Max Daniel Kauther

**Affiliations:** Klinik für Unfall-, Hand- und Wiederherstellungschirurgie, University Hospital Essen, Hufelandstraße 55, 45147 Essen, Germany

## Abstract

We describe the case of a 45-year-old woman who suffered an impalement injury of the pelvis with penetration of the sciatic foramen by a wooden foreign body. Following a single operation, the injury healed without complications or infection. We have taken this as an opportunity to describe the case and our standard procedure in more detail.

## 1. Introduction

Before we started writing this report, we chose the terms “penetrating pelvic trauma” and “pelvic impalement” for our literature research.

For the former, we obtained 12 results, mostly describing penetrating injuries due to metallic foreign bodies. Five articles reported on gunshot wounds, and about half of the results reported on trauma in a military context with appropriate treatment strategies and options.

The search term “pelvic impalement” yielded four results, only one of which explicitly referred to wooden impalement trauma [1].

Pelvic trauma is a common occurrence and is mainly associated with traffic accidents. An evaluation of the German DGU TR data from 2015 to 2017 showed that 95% of all trauma patients suffered blunt trauma, while 15% had a pelvic injury [2]. In addition to the frequent blunt pelvic injuries, penetrating and especially impaling pelvic injuries are clearly underrepresented. For penetrating pelvic injuries in the military context, mortality of between 21% and 36% is stated in the literature [3]. Besides the general risks resulting from penetrating traumas, those affecting the pelvic region involve the additional high risk of infection due to possible injury to the rectum or colon. If hypotension occurs at the same time and colonoscopy cannot be performed, mortality increases significantly [4].

The anatomical conditions in the pelvis make injuries to important vessels or nerves very likely in penetrating pelvic injuries. Significant bleeding, as can occur with both blunt and penetrating trauma in the pelvic area, causes high mortality and morbidity [5]. Pelvic trauma is common in emergency medicine, especially injuries caused by high-speed violence. The significance of the case we describe here lies in the accident mechanism and in the material of the penetrating foreign body.

However only a few cases of pelvic injuries caused by the penetration of a wooden foreign body have been reported in the literature so far. This small number encouraged us to publish this case report in order to increase the available documentation concerning treatment of these injuries.

## 2. Case Report

We report on a 45-year-old female patient who sustained a pelvis penetrating injury after a fall from a height of three meters. The patient fell while working on a ladder and landed with the right gluteal region on a wooden fence. The portal of entry of the wooden fence pole was located laterally at the junction of the gluteal region and the dorsolateral thigh. Passers-by rescued the victim by lifting her off the fence pole. When the ambulance arrived, the patient was conscious, oriented, and in a hemodynamically stable condition. Circulation, motor functions, and sensitivity in all extremities were intact. In the initial body check, instability of the pelvis could not definitely be ruled out. The recap time of the legs was 3-4 seconds. Following the establishment of two large lumen peripheral accesses, analgosedation of the patient (height: 166 cm; weight: 70 kg) with a total of 30 mg esketamin and 3 mg midazolam was performed. After approximately 20 minutes of treatment on-site with appropriate salvage recovery and stabilization of the pelvis by means of a pelvic sling, the patient was taken to the Trauma Surgery Department of the University Hospital Essen and admitted to the shock room.

On admission, the patient was responsive (GCS 13) and hemodynamically stable (HR: 62/min; BP: 109/62 mmHg; RR: 12/min; SpO_2_: 99%; shock index: 0.6; Revised Trauma Score: 8). Clinical examination of the patient in the shock room revealed a moderately bleeding wound in the area of the right gluteal region of about 5 cm in diameter. Instability of the pelvis could not be excluded with certainty. The initial Hb was 13.1 g/dl. Parallel to the clinical examination, FAST sonography was performed and yielded no evidence of free intraabdominal fluid.

**Figure 1:**
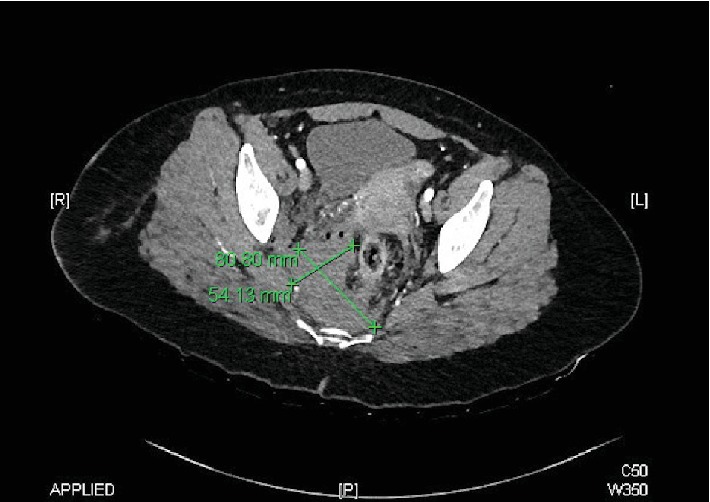
Presacral hematoma in the pelvic CT.

**Figure 2:**
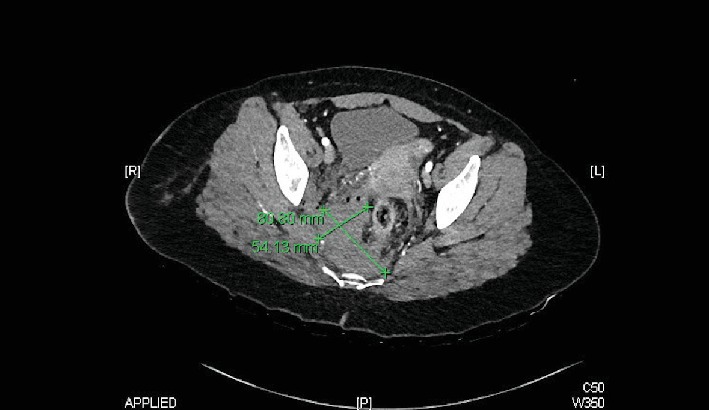
Hematoma in pelvic CT.

While still in the shock room, the patient received antibiotic therapy according to our internal guidelines with cefazolin 2 g. This was followed by an X-ray of the pelvis and the right thigh. A subsequent CT body scan revealed no evidence of active arterial bleeding or foreign body involvement in the pelvic area or any other relevant injuries, but did show a right-sided presacral hematoma with multiple fine air pockets on the right side (Figures 1 and 2). The rectum was displaced towards ventral and left lateral. In the perirectal adipose tissue, several fine air inclusions were delineated in the right lateral pelvic region, and a penetration canal through the M. gluteus maximus was visible (Figures 1 and 2). The veins and arteries were visualized very clearly with good contrast. There were no signs of free fluid and therefore no reason to suspect acute bleeding. There was also no free intraabdominal air. The patient had no history of previous illnesses or permanent medication.

Emergency surgery was performed immediately. Anesthesia was induced by rapid sequence induction with fentanyl, propofol, and rocuronium and maintained using isoflurane as ITN. As mentioned above perioperatively, the patient received cefazolin 2 g i.v. as a single shot. Since no intraabdominal fluid was visible in the radiological diagnosis, a colonoscopy was performed first because of suspected rectal perforation.

**Figure 3:**
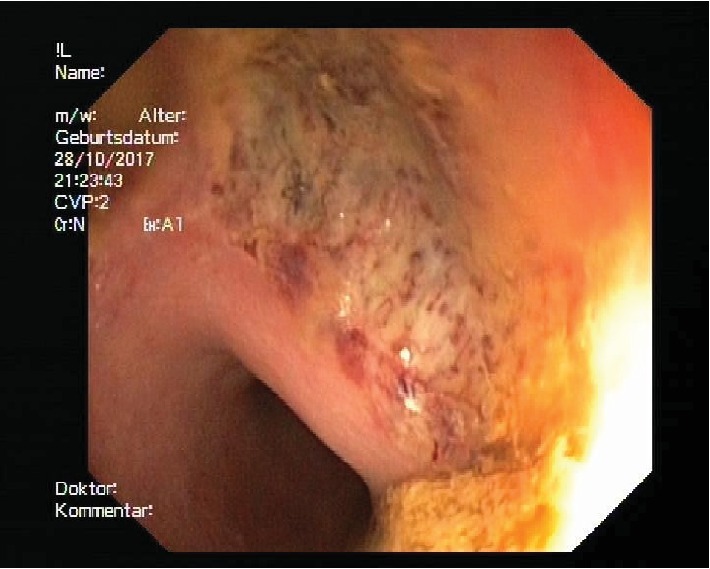
Hematoma in colonoscopy.

**Figure 4:**
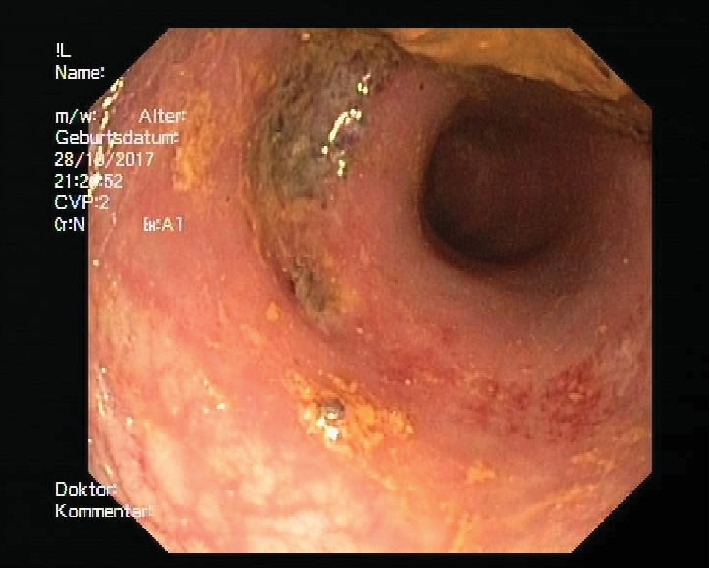
Hematoma in colonoscopy.

A total of 40 cm from anal were seen. At 10 cm from anal there was a circular erythematous, fibrin-occupied ulceration with blood spots without the evidence of perforation (Figures 3 and 4).

Subsequently, exploration of the wound was commenced.

The wound edges were excised, and the wound was enlarged in longitudinal section to 7 cm in order to allow safe exploration of the wound bed.

Examination of the wound canal revealed that the gluteus maximus muscle was perforated and there was a lesion of the gluteus medius muscle at the margins.

A coagulated hematoma was removed by careful and repeated irrigation of the perforation channel with constant palpatory control.

The perforation canal could be palpated through the foramen ischiadicum as far as the area of the rectum. A further coagulated hematoma was found in the area of the foramen ischiadicum. Here, there was no indication of active bleeding.

Apart from the muscular lesions, there was no evidence of injury to any of the pathways of the vascular and nerval tracts.

Under palpatory control, a size-16 Charrière Redon drain was inserted and repeated careful and extensive irrigation of the penetration canal was carried out. No splinters of the foreign body were detected, either by palpation or by irrigation.

A second Redon drain, Charrière 12, was introduced subcutaneously after closure of the gluteal fascia.

The wound was closed by dermal suture with inserted drains (Prolene 2.0).

Postoperatively, the patient was extubated promptly.

The blood flow and the motor and sensory system of the right leg was always intact during inpatient treatment.

Postoperatively, the patient received piperacillin/tazobactam 4.5 g three times daily for six days i.v.

Further treatment in our IMCU was carried out for five days. The drains did not collect any relevant amount of secretion or blood and were removed four days postoperatively.

Initially, the patient was mobilized with partial weight-bearing and from the eighth postoperative day pain-adapted full weight-bearing was permitted.

Thrombosis prophylaxis was performed with enoxaparin-natrium 40 mg once daily until safe full weight-bearing was possible.

The patient remained in the hospital for 12 days postoperatively.

Approximately two and a half weeks after the trauma, an out-patient follow-up colonoscopy was performed. Here, the ulcerations described above had left a scar which presented no complications.

The patient returned for out-patient follow-up examination seven months after discharge.

Using VAS, the patient reported the following pain intensities for the respective time points: immediately after trauma—1; shock room—2; immediately post op—6; two days post op—3; and 8 months after trauma—1.

In an evaluation according to the SF36 score, the patient reached 95% for physical function seven months after trauma.

The range of motion of the hips at that time is as follows: Ext/Flex left—10-0-110; Ext/Flex right—10-0-110; ER/IR left—40-0-30; ER/IR right—40-0-30; Abd/Add left—45-0-30; and Abd/Add right—30-0-15. The scar had healed without irritation.

Overall, follow-up examination revealed a favorable long-term outcome without any residual effects which would limit the patient's quality of life. Only the range of abduction and adduction in the right hip appeared to be reduced in comparison with the contralateral hip. The patient's gait was normal.

## 3. Discussion

We consider the following points to be crucial for the treatment of an impaling injury in the pelvic area.

In the preclinical phase, the procedure described in the PTLS recommendations for blunt pelvic trauma should be followed, since in this setting the exact nature and extent of the injury cannot be sufficiently differentiated [6].

In the first clinical phase, we consider the procedure according to the ATLS to be the best strategy.

Hornez et al. in 2016 [7] designed a flowchart for the treatment of penetrating injuries.

While we largely agree with this chart, we would like to add a few points. First, when a hemodynamically unstable patient is delivered to the emergency department, the procedure according to the principles of damage control is without alternative [7]. The hemodynamically unstable patient is at a great risk of dying and all attention must be focused on preventing death by stopping the loss of blood. With hemodynamically stable patients, in contrast, there is more time to focus on the long-term outcome and the possibility of restitutio ad integrum. Second, in addition to standard diagnostics (X-ray, CT angiography), the foreign body itself should also be at the center of attention. The already high risk of infection due to penetrating injuries should not be unnecessarily increased by the fact that the properties of the foreign body are not taken into account. Metal has a high radiopacity, but a wooden foreign object is insufficiently visualized by X-ray diagnostics. Due to the nature of wood, there is a high risk of splintering or incomplete recovery of the foreign body. For wooden foreign bodies, a maximum sensitivity of 77.2% can be assumed in radiological CT diagnosis [8]. In our opinion, this weakness in imaging diagnostics of wooden foreign bodies must be compensated by thorough and comprehensive surgical exploration. Even with a negative result from imaging diagnostics, the surgical procedure should always be chosen so as not to overlook fragments of a foreign body and thereby risk an unnecessary foreign body-associated infection [9].

We therefore see the necessity for larger studies with more patients to develop more advanced and differentiated concepts for the treatment of penetrating pelvic trauma by wooden foreign objects.

## 4. Conclusions

- *Preclinical*: treatment according to the recommendations for polytrauma or blunt pelvic trauma.
- *Emergency department*: treatment according to ATLS principles and rapid differentiation between vitally vulnerable patients and vitally less endangered patients.
  - Vitally endangered patients: immediate surgical treatment according to damage control principles and hemodynamic stabilization [7].
  - Vitally less endangered patients: rapid but extensive preoperative diagnosis (X-ray, CT angiography, and if necessary, sonography) and calculated preoperative antibiotic therapy (amoxicillin/clavulan acid 1000/2000 mg+metronidazole 500 mg i.v.). Surgical removal of the foreign body with extensive surgical exploration, microbiological samples, debridement, local antiseptic therapy, drainage, and continuation of the preoperatively commenced antibiotic therapy until results of the microbiological samples are available.
